# Inflammatory Neuropathy of the Lumbosacral Plexus following Periacetabular Osteotomy

**DOI:** 10.1155/2016/3632654

**Published:** 2016-02-16

**Authors:** Stijn Ghijselings, Frans Bruyninckx, Hendrik Delport, Kristoff Corten

**Affiliations:** ^1^UZ Pellenberg Orthopedic Department, University Hospitals Leuven, Weligerveld 1, 3212 Pellenberg, Belgium; ^2^UZ Pellenberg Department of Physical Medicine and Rehabilitation, University Hospitals Leuven, Weligerveld 1, 3212 Pellenberg, Belgium; ^3^Department of Orthopedics, Ziekenhuis Oost-Limburg, Schiepse Bos 6, 360 Genk, Belgium

## Abstract

*Introduction*. During periacetabular osteotomy (PAO), the sciatic, femoral, and obturator nerves are at risk. Most frequently nerve lesions can be attributed to a mechanical cause; however, in the absence of a clear mechanical cause surgeons are faced with a diagnostic problem and in many cases no diagnosis will be established. We report a case of inflammatory neuropathy of the lumbosacral plexus following a PAO.* Case Presentation. *A 31-year-old female developed weakness of ankle and knee flexion and extension 6 months after a PAO. Electrophysiological studies revealed damage to the obturator, femoral, and sciatic nerve consistent with an inflammatory lumbosacral plexopathy. MRI of the lumbosacral plexus was normal. The patient was treated with multimodal pain therapy and prolonged physiotherapy; nevertheless, symptoms worsened over time. At 2-year follow-up, there were no signs of recovery.* Discussion. *Inflammatory neuropathy of the lumbosacral plexus is a potential cause of pain and weakness after ipsilateral orthopaedic procedures. It should be distinguished from more frequently encountered mechanical causes of postsurgical neuropathy based on clinical suspicion, electrophysiological studies, MRI, and nerve biopsy. It is important that the orthopaedic community is aware of this complication since there is some evidence that early recognition and initiation of immunosuppressive therapy can lead to improved clinical outcome.

## 1. Introduction

Peripheral nerve damage after hip surgery is a devastating complication. During periacetabular osteotomy (PAO), the sciatic, femoral, obturator, and lateral femoral cutaneous nerves are at risk. Most frequently, these nerve lesions can be attributed to a mechanical cause, which happened either during or shortly after surgery. However, in the absence of a clear mechanical cause, surgeons and anesthesiologists are faced with a diagnostic problem and in many cases no diagnosis will be established. A wait-and-see strategy is often adopted, hoping for the symptoms to improve spontaneously. Recently an inflammatory etiology was found to be the cause of unexplained postsurgical neuropathy in a number of cases. In the upper limb, this (idiopathic) inflammatory plexopathy is much more common and known as Parsonage-Turner syndrome. In the lower limb, only a few cases of postsurgical inflammatory neuropathy were described. We report a case of inflammatory neuropathy of the lumbosacral plexus following a PAO. This condition is underrecognized and should be distinguished from more frequently encountered mechanical causes of postsurgical neuropathy since there is some evidence that early recognition and initiation of immunosuppressive therapy can lead to improved clinical outcome.

## 2. Case Presentation

**Figure 1:**
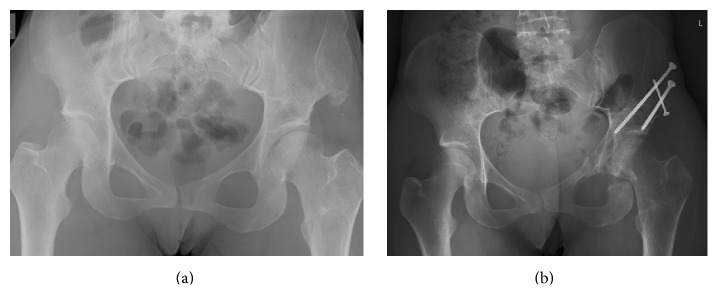
(a) AP pelvic X-ray demonstrating acetabular dysplasia of the left hip. (b) Normalisation of the CEA and healing of the osteotomy sites 3 months after periacetabular osteotomy.

A 31-year-old female with a history of developmental dysplasia of the left hip presented with chronic pain in the left hip and knee. She was a smoker and her BMI was 17.3 kg/m^2^. During childhood, she had undergone several procedures to the hip including a Salter osteotomy. Radiographic evaluation demonstrated a lateral center edge angle (CEA) of 18° and an anterior CEA of 5° (Figure 1). Arthro-CT showed an intact cartilage layer and an MRI of the knee was normal. She was initially treated with intra-articular corticoid injections with short-term benefit. However, after 2 years of conservative treatment, it was decided to perform a Ganz PAO to improve acetabular coverage. The surgery was performed under general anesthesia and patient controlled epidural analgesia was prescribed for postoperative pain control. The patient received a transfusion with 2 units of packed red cells because of postoperative anemia. Immediately after surgery, she suffered from hypoesthesia in the groin and mild adductor weakness due to neurapraxia of the obturator nerve. Clinical control at 3 months showed improvement of the obturator nerve neurapraxia and a normal function of the femoral and sciatic nerve. Radiographs demonstrated healing of the osteotomy sites and a normal CEA (Figure 1). Electromyography revealed signs of ongoing reinnervation in the obturator nerve distribution and a reduced contraction pattern in the femoral and sciatic nerve musculature. Full weight bearing was allowed and physiotherapy was continued. However, at 6 months postoperatively, she complained of increasing pain and weakness in the left leg and the inability to walk without support. Clinical examination demonstrated weakness of ankle and knee flexion and extension, significant atrophy of the quadriceps muscle, and a colder and blue discolored left leg. Straight leg raise was impossible and there was still remaining hypoesthesia in the groin. Electrophysiological studies revealed severe damage to the obturator nerve, the femoral nerve, and the sciatic nerve consistent with an inflammatory postsurgical lumbosacral plexopathy. MRI of the lumbosacral plexus did not reveal any abnormal findings. The patient was treated with multimodal pain therapy and prolonged physiotherapy; nevertheless, symptoms worsened over time. At 2-year follow-up, there were no signs of recovery and she was only able to walk short distances using 2 crutches.

## 3. Discussion

Postsurgical neuropathy is a rare complication after hip surgery and can often be attributed to mechanical factors such as stretch, compression, contusion, hematoma, or nerve transection. Careful surgical technique and attention to patient positioning are key in preventing this complication. In the presence of an obvious correctable cause (hematoma, nerve transection), surgical exploration can solve the problem, but in most cases little more can be done than to wait for spontaneous improvement of the symptoms.

Recently the role of a primary inflammatory process as the etiology for postsurgical neuropathy has been described. Staff et al. described the characteristics of 21 patients with biopsy-confirmed postsurgical inflammatory neuropathy [1]. Nerve biopsies taken distal from the site of surgery showed evidence of ischaemic nerve injury and an increased perivascular lymphocytic inflammation suggestive of microvasculitis. Although the cause of this inflammatory neuropathy is far from clear, possible contributing factors are the surgical process, transfusions, and anaesthetics. Other possible risk factors reported include diabetes mellitus, cancer, infection, and a history of smoking. In this series, 5 patients developed neuropathy in the same lower limb where they had undergone an orthopaedic procedure. Based on these cases, the authors concluded that there is strong evidence for an inflammatory component in some cases of neuropathy after orthopaedic procedures in the lower limb.

Laughlin et al. reported 7 patients with ipsilateral weakness and pain after hip surgery (THA = 5, PAO = 1, and femoral nail = 1) due to an inflammatory neuropathy including 4 cases previously published by Staff et al. [2]. Clinical criteria for diagnosis were onset in the immediate postoperative period, progression of weakness or pain postoperatively, weakness outside of the distal sciatic nerve distribution, and no improvement in weakness or pain within the first postoperative month. MRI of the lumbosacral plexus is not diagnostic but is essential to exclude other causes of neuropathy such as nerve compression or transection. Electrophysiological testing showed findings consistent with axonal damage in all patients. Nerve involvement was frequently patchy, a pattern often seen in inflammatory neuropathies. All patients underwent nerve biopsy demonstrating inflammatory neuropathy diagnostic of nerve microvasculitis, consistent with results reported by Staff et al. Six (of 7) patients were treated with intravenous methylprednisolone (1 g/wk for 12 weeks). All patients had improvement of their impairment and pain scores to a larger extent than the 1 untreated patient.

In another case series, Rattananan et al. treated 5 patients with postsurgical inflammatory neuropathy with intravenous methylprednisolone (1 g/wk for 12 weeks). Four patients had an improvement of pain and weakness after 12 weeks, but no improvement was seen in one patient. In this patient, therapy was initiated more than 2 years after the neuropathy onset, while this was within one year after onset in the other patients. Based on their experience, the authors recommend to start treatment as soon as possible to decrease and prevent further neurological worsening [3].

Inflammatory neuropathy is much more common in the upper limb and known as the Parsonage-Turner syndrome or neuralgic amyotrophy (NA). Van Alfen and Van Engelen reported an antecedent event in up to 53.2% of the cases, which was a surgical procedure in 13.9%. In most cases, NA occurs as a sporadic disorder, but occasionally an autosomal dominant hereditary trait is present. In hereditary, NA attacks occur at a younger age and the recurrence rate is higher. Interestingly nerves outside the brachial plexus are more frequently affected and in 32.6% of the attacks the lumbosacral plexus is involved [4]. The trigger for developing inflammatory neuropathy after surgery is far from clear but a combination of stress responses from surgery, a genetic predisposition, preexisting neuropathy, and mechanical forces is likely [1, 5].

In summary, inflammatory neuropathy of the lumbosacral plexus is a potential cause of pain and weakness of the lower limb after ipsilateral orthopaedic procedures. This condition is underrecognized and potentially treatable. It should be distinguished from more frequently encountered mechanical causes of postsurgical neuropathy based on clinical suspicion, electrophysiological studies, MRI of the lumbosacral plexus, and nerve biopsy. There is some evidence that early recognition and initiation of immunosuppressive therapy can lead to improved clinical outcome.
